# Heat Shock Protein 27 Is an Emerging Predictor of Contrast-Induced Acute Kidney Injury on Patients Subjected to Percutaneous Coronary Interventions

**DOI:** 10.3390/cells10030684

**Published:** 2021-03-19

**Authors:** Andrzej Jaroszyński, Tomasz Zaborowski, Stanisław Głuszek, Tomasz Zapolski, Marcin Sadowski, Wojciech Załuska, Anna Cedro, Teresa Małecka-Massalska, Wojciech Dąbrowski

**Affiliations:** 1 Collegium Medicum, Jan Kochanowski University in Kielce, 25-317 Kielce, Poland; sgluszek@wp.pl (S.G.); msadowski@ujk.edu.pl (M.S.); 2 Department of Nephrology, Wojewódzki Szpital Zespolony in Kielce, 25-736 Kielce, Poland; 3 Department of Family Medicine, Medical University of Lublin, 20-954 Lublin, Poland; tzaborowski@wp.pl; 4 Department of Cardiology, Medical University of Lublin, 20-954 Lublin, Poland; zapolia@wp.pl; 5 Department of Nephrology, Medical University of Lublin, 20-954 Lublin, Poland; wtzaluska2@poczta.onet.pl; 6 The Institute of Public Health, Jan Kochanowski University in Kielce, 25-317 Kielce, Poland; aniacedro@op.pl; 7 Department of Human Physiology, Medical University of Lublin, 20-080 Lublin, Poland; teresa.malecka-massalska@umlub.pl; 8 Department of Anesthesiology and Intensive Care, Medical University of Lublin, 20-954 Lublin, Poland; w.dabrowski5@gmail.com

**Keywords:** heat shock protein 27, acute kidney injury, contrast-induced nephropathy, percutaneous coronary interventions

## Abstract

Contrast-induced acute kidney injury (CI-AKI) is a serious complication associated with considerable morbidity and mortality. Heat-shock protein 27 (HSP27) plays a role in the defense of the kidney tissue against various forms of cellular stress, including hypoxia and oxydative stress, both features associated with CI-AKI. The aim of our study was to evaluate a potential predictive value of HSP27 for CI-AKI in patients subjected to percutaneous coronary interventions (PCI). Included were 343 selected patients subjected to PCI. Exclusion criteria were conditions that potentially might influence HSP27 levels. HSP27 serum levels were evaluated prior to PCI, together with serum creatinine, the concentration of which was also evaluated twice at 48 and 72 h post PCI. CI-AKI was diagnosed in 9.3% of patients. Patients in whom CI-AKI was diagnosed were older (*p* < 0.001), were more often females (*p* = 0.021), had higher prevalence of diabetes (*p* = 0.011), hypotension during PCI (*p* < 0.001), albuminuria (*p* = 0.004) as well as multivessel disease (*p* = 0.002), received higher contrast volume (*p* = 0.006), more often received contrast volume (CV) above the maximum allowed contrast dose (MACD) (*p* < 0.001), and had lower HSP27 level (*p* < 0.001). On multivariate analysis, CV > MACD (OR 1.23, *p* = 0.001), number of diseased vessels (OR 1.27, *p* = 0.006), and HSP27 (OR 0.81, *p* = 0.001) remained independent predictors of CI-AKI. Low concentration of HSP27 is an emerging, strong and independent predictor of CI-AKI in patients subjected to PCI.

## 1. Introduction

Contrast-induced acute kidney injury (CI-AKI) following the use of iodine-based radiographic contrast media (RCM) for diagnostic as well as therapeutic procedures is the third common cause of hospital-acquired AKI. CI-AKI is a serious complication associated with considerable morbidity and mortality. The incidence of CI-AKI is significant, and CI-AKI remains a concern for patients undergoing percutaneous coronary interventions (PCI) [1,2,3,4].

The mechanisms underlying the CI-AKI have not been completely understood. The growing evidence exists, however, that ischemia reperfusion injury (IRI), hypoxia, and increased oxidative stress play a significant role in the CI-AKI pathogenesis [1,2,3,5,6,7].

Heat-shock proteins (HSPs) are a protein superfamily whose presence is found in all cells of all organisms. HSPs are synthesized in response to various nociceptive factors and protect cells against different forms of cellular stress, including oxidative stress, as well as apoptosis. HSPs play an important role in the intra- or extracellular defense of the kidney tissue. Heat-shock protein 27 (HSP27) is a member of the small molecular weight HSP family. High levels of HSP27 are normally present in the renal medulla in response to conditions of hypoxia and oxydative stress. Evidence exists that HSP27 prevents injury and restores normal cellular function in the kidney following IRI [8,9,10,11,12,13,14,15]. The association between HSP27 and CI-AKI, however, has not been elucidated.

Owing to the role HSP27 plays in the kidneys and that IRI and oxidative stress are characteristic features of CI-AKI, we hypothesized that HSP27 levels might have a predictive value for CI-AKI risk in patients subjected to PCI.

## 2. Methods

### 2.1. Study Population

Included in the study were 343 selected patients subjected to PCI (168 women and 175 men), aged 50–70 years (mean 66.36 ± 7.32). The following exclusion criteria were applied: age < 50 years and > 70 years (to minimize the potential influence of age on HSP27 levels), eGFR < 45 mL/min, myocardial infarction during the last 3 months, left ventricular ejection fraction (LVEF) < 50%, neoplasma, C-reactive protein > 10 mg/dL (to avoid the influence of chronic kidney disease, myocardial infarction, heart failure, neoplasma and inflammation on HSP27 concentrations), and thyroid diseases. Excluded were also patients who received renal toxic medicine or RCM for any reason during the previous 2 months. Owing to the fact that it was impossible to estimate population size meeting the criteria used in our study, the sample size calculation was not performed. Included were all available patients. 

### 2.2. Percutaneous Coronary Interventions

Patients with stable angina pectoris were subjected to elective PCI based on indications determined by the result of coronary angiography, which was performed at least 2 months earlier (to minimize the influence of RCM during coronary angiography). PCI was performed using standard techniques by the operator who decided on the type of the stents used as well as the approach site. Nonionic, low-osmolality RCM was used in all cases. For the prevention of CI-AKI, isotonic saline was administered intravenously in all patients according to guidelines (1.0–1.5 mL/kg/h started 12 h before PCI and continued up to 24 h after PCI) [16]. Maximum allowed contrast dose (MACD) was calculated for each patient using the following formula: [5 mL of contrast/kg body weight (maximum 300 mL)]/serum creatinine (mg/dL). Then, the number of patients who received contrast volume (CV) in the dose exceeding MACD was calculated. Hypotension was diagnosed when systolic blood pressure was <80 mmHg and/or the patient required the treatment with inotropes or saline infusion was needed when PCI was performed.

### 2.3. Acute Kidney Injury Assessment

Serum creatinine (SCr) was determined pre-PCI and then twice at 48 and 72 h. CI-AKI was diagnosed using the change between peak SCr level and pre-PCI value. CI-AKI was defined and classified into 3 grades according to the Acute Kidney Injury Network (AKIN) criteria [17]. eGFR was calculated using the CKD-EPI formula.

### 2.4. Clinical Data and Biochemical Measurements

Commercially available ELISA kits (Invitrogen eBioscience Human HSP27 Platinum, Product Code 15541607) were used to determine serum contents of HSP27 according to the manufacturer’s protocols. Baseline demographic, and laboratory as well as clinical data were also collected.

### 2.5. Statistical Analysis

Statistical analysis was performed using Statistica Version 10 as described in detail previously [14]. The statistical significance of the differences between pre- and post-PCI results were compared using Student’s *t*-test or using the Mann–Whitney U-test, when appropriate. Linear regression analysis was performed using the Pearson or Spearman test, as appropriate. For CI-AKI risk factors analysis, patients were divided into two groups according to the presence of CI-AKI (CI-AKI group and non CI-AKI group). Univariate as well as multivariate logistic regression analysis was used to estimate odds ratio (OR) of associated risk factors for RCM-induced CI-AKI. Variables showing a *p*-value < 0.05 in univariate analysis entered the model. The receiver operating characteristics curves (ROC) were performed to determine optimal cut-off points for HSP27 in predicting CIN. Probability values of *p* < 0.05 were accepted as significant.

## 3. Results

### 3.1. Baseline Characteristics

Clinical characteristics of the studied patients are summarized in Table 1.

### 3.2. The Influence of Radiographic Contrast Media on Serum Creatinine Levels

RCM-induced SCr increase associated with PCI procedure was found in 207 (60.4%) patients, in 61 (17.8%) patients SCr was unchanged, whereas in 75 (21.9%) Scr decreased post-PCI. CI-AKI was diagnosed in 32 (9.3%) patients (grade 1 in 21, grade 2 in 8 patients, and grade 3 in 3 patients). Mean SCr increase in CI-AKI group was 0.51 ± 0.39 mg/dl. None of the CI-AKI patients required renal replacement treatment. CV > MACD was found in 20.1% of patients.

### 3.3. Differences between Patients with and without CI-AKI

Patients in whom CI-AKI was diagnosed were older (*p* < 0.001), were more often females (*p* = 0.021), had a higher prevalence of diabetes (*p* = 0.011), had hypotension during PCI (*p* < 0.001), albuminuria (*p* = 0.004), received CV > MACD (*p* < 0.001), had a higher number of diseased vessels (*p* = 0.002), higher RCM volume (*p* = 0.006), and had lower HSP27 level (*p* < 0.001) (Table 1). No significant differences between CI-AKI and non CI-AKI groups were found in the prevalence of hypertension and prior myocardial infarction as well as smoking. Similarly, no differences were observed in lipids, medication and ejection fraction between CI-AKI and non CI-AKI groups. No difference was found in baseline eGFR between CI-AKI and non CI-AKI groups (*p* = 0.114). The correlation between eGFR and HSP27 was also not significant (*r* = 0.108; *p* = 0.099).

### 3.4. Univariate and Multivariate Analyses for the Prediction for CI-AKI

Table 2 presents analyses for the prediction for CI-AKI. On univariate analysis, age (OR 1.21, *p* < 0.001), female gender (OR 1.29, *p* = 0.021), diabetes mellitus (OR 1.09, *p* = 0.016), ejection fraction (OR 0.85, *p* = 0.009), hemoglobin (OR 0.89, *p* = 0.022), hypotension during PCI (OR 1.21, *p* = 0.013), CV > MACD (OR 1.37, *p* < 0.001), number of diseased vessels (OR 1.31, *p* = 0.006), albuminuria (OR 1.11, *p* = 0.014), and serum HSP27 (OR 0.78, *p* < 0.001) were predictors of CI-AKI. On multivariate analysis, CV > MACD (OR 1.23, *p* = 0.001), number of diseased vessels (OR 1.27, *p* = 0.008), and HSP27 (OR 0.81, *p* = 0.001) remained independent predictors of CI-AKI.

### 3.5. ROC Analysis

**Figure 1:**
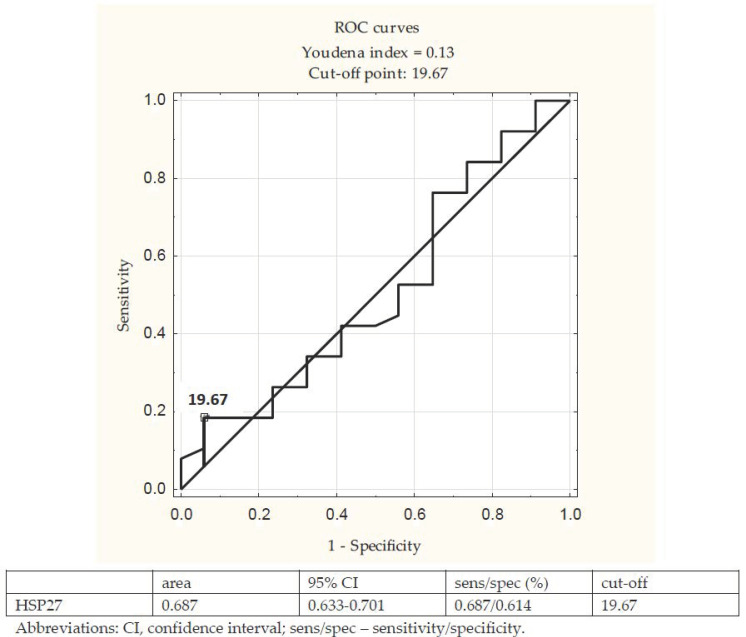
Receiver operating characteristics (ROC) curve of heat-shock protein 27 (HSP27) in predicting CI-AKI.

The ROC analysis of HSP27 as a predictor of the CI-AKI was performed. The analysis showed the AUC equal to 0.687 and the sensitivity/specificity equal to 0.687/0.614. The cut-off point was 19.67 µg/L. The ROC curves are presented in Figure 1.

## 4. Discussion

The main finding of the current study is that low HSP27 level is a strong and independent predictor of CI-AKI in patients subjected to PCI. Additionally our study confirmed that the incidence of CI-AKI is significant even in relatively low-risk patients.

The incidence of CI-AKI due to PCI varies in a wide range depending on the diagnostic criteria, associated risk factors, concomitant diseases as well as applied prevention strategy [7,18,19,20]. In a large population of patients undergoing PCI, the incidence of CI-AKI was 7.1% in all-comers [1]; however, the risk increases to even 50% in patients with multiple risk factors [21]. In our study the incidence of CI-AKI was 9.3% and stage 1 of CIN was the most frequently observed degree of CI-AKI. Given that only stable patients were qualified to our study, the incidence of CI-AKI was relatively high compared to other results [1,4]. This may be due to the high prevalence of diabetes as well as patients with multivessel disease in the studied population. However, our results are similar to those obtained by Demir et al. [22], who used the same diagnostic criteria as well as prevention protocol.

To our knowledge this is the first study that revealed that low HSP27 serum levels predict CI-AKI in PCI patients. Mechanisms responsible for CI-AKI have not been fully elucidated; however, two mechanisms play a main role. After the initial increase in renal blood flow, the RCMs induce prolonged vasoconstriction, enchance vascular resistance and reduce renal blood flow, leading to hypoxia, increased oxidative stress, and ischemia reperfusion injury (IRI). Additionally, the RCMs exert direct toxic effects on both endothelial and tubular cells, finally resulting in cell damage, apoptosis, and cell death. Moreover, both processes can aggravate each other [2,3,7,8].

Given that in the present study low HSP27 levels were associated with CI-AKI, we can speculate that HSP27 may exert a protective effect against CI-AKI. It is in agreement with previous reports on this issue. It has been demonstrated that HSPs, including HSP27, exert cytoprotective effects, interact with other proteins to facilitate normal cellular functions, take part in repairing proteins, target damaged protein for degradation, inhibit apoptosis, and exhibit antioxidant properties. HSP27 plays a crucial role in reducing the cell injury as well as protecting cells against different forms of cellular stress, including hypoxia and oxidative stress, both features associated with CI-AKI [6,13,23,24,25,26,27,28]. In physiological conditions HSP27 is present at the highest levels in renal medulla [8,29]. Even in normal conditions oxygen delivery to renal medulla is poor, thus vasoconstriction due to CI-AKI causes particularly severe consequences. It was demonstrated in animal models that acute ischemic kidney damage induces a 12-fold increase of HSP27 expression in the kidney tissues with a peak level observed 6 h post-reperfusion [12]. It suggests a protective role of HSP27 against hypoxia and oxidative stress [8,9,10,12,15,25]. The evidence exists that this chaperone protein inhibits apoptosis in IRI models [7]. Moreover, Kim et al. [29] demonstrated that HSP27 provides renal protection against IRI, whereas Fujigaki et al. showed that HSP27 prevents toxin-induced kidney injury [30]. Since HSP27 potentially prevents all main processes involved in CI-AKI pathogenesis, it can be hypothesized that low HSP27 serum levels may indirectly reflect low HSP27 concentrations in the kidney tissue, explaining our results. Further studies are required to confirm our results as well as to determine if serum HSP27 is only a marker of CI-AKI risk or whether it identifies more distinct mechanisms and can be a potential therapeutic target.

The present study has some important limitations. The first limitation is that our patients are selected. It limits factors that potentially influence HSP27 concentrations, but makes the study less generalizable. The second limitation is that our study is merely descriptive, and a pathophysiologic explanation for the relation between HSP27 and CI-AKI needs to be part of further studies. Finally, given that it is a preliminary study, we evaluated HSP27 levels exclusively pre-PCI and it is likely that serial rather than single measurements of HSP27 may influence the results.

## 5. Conclusions

Low concentration of HSP27 is an emerging, strong and independent predictor of CI-AKI in patients subjected to PCI.

## Figures and Tables

**Table 1:** Differences in baseline characteristic between patients with and without contrast-induced acute kidney injury (CI-AKI).

Parameter	All Patients *n* = 343	CI-AKI *n* = 32	Non CI-AKI *n* = 311	*p*
Age (years)	66.36 ± 7.32	72.8 ± 6.72	64.4 ± 6.95	<0.001
Female gender (%)	38.2	46.9	37.3	0.021
Prior MI (%)	25.9	27.3	25.8	0.496
Diabete smellitus (%)	48.0	63.4	45.1	0.011
Hypertension (%)	83.7	81.1	83.9	0.504
Smoking (%)	18.1	17.8	18.2	0.687
EF (%)	54.83 ± 5.33	52.70 ± 5.21	55.14 ± 5.56	0.165
Hemoglobin (g/dL)	11.68 ± 1.09	10.43 ± 1.08	11.98 ±1.02	0.091
Total cholesterol (mg/dL)	188.3 ± 37.35	188.2 ± 35.15	188.3 ± 37.35	0.674
LDL cholesterol (mg/dL)	114.8 ± 30.11	116.2 ± 29.19	114.7 ± 29.08	0.544
HDL cholesterol (mg/dL)	43.82 ± 17.08	43.41 ± 16.89	43.85 ± 16.45	0.715
Triglycerides (mg/dL)	176.3 ± 59.27	170.2 ± 59.03	176.6 ± 58.9	0.314
Hypotension during PCI (%)	9.5	22.7	8.1	<0.001
RCM volume (mL)	179 ± 58	198 ± 41	175.2 ± 52	0.006
CV > MACD (%)	20.1	28.1	19.4	<0.001
Number of diseased vessels (*n*)	2.3 ± 0.7	2.6 ± 0.8	2.2 ± 0.7	0.002
eGFR (mL/min/1.73 m^2^)	62.7 ± 9.04	59.9 ± 8.31	62.90 ± 8.91	0.114
Albuminuria (%)	18.1	25.0	17.7	0.004
HSP27 (µg/L)	32.7 ± 8.14	21.19 ± 7.87	35.3 ± 8.11	<0.001
ACE/ARB (%)	86.0	87.5	85.6	0.327
Beta blockers (%)	75.8	78.1	74.9	0.213
Statins (%)	82.2	81.3	82.90	0.614
Diuretics (%)	19.0	18.8	19.1	0.799

Abbreviations: MI, myocardial infarction; EF, ejection fraction; PCI, percutaneous coronary interventions; RCM, radiographic contrast media; MACD, maximum allowed contrast dose; ACE/ARB, angiotensin-converting enzyme inhibitors/angiotensin receptor blockers; HSP27, heat shock protein 27.

**Table 2:** Predictors of contrast-induced nephropathy.

Parameter	Univariate OR (95% CI)	*p*	Multivariate OR (95% CI)	*p*
Age (years)	1.31 (1.07–1.1.58)	<0.001	1.18 (0.98–1.92)	0.104
Female gender (%)	1.26 (0.98–2.17)	0.021	1.23 (0.79–2.31)	0.113
Prior MI (%)	1.12 (0.64–2.23)	0.187		
Diabete mellitus (%)	1.09 (0.73–1.549	0.016	1.11 (0.63–1.83)	0.247
Hypertension (%)	1.13 (0.73–2.051)	0.285		
Smoking (%)	1.35 (0.77–2.87)	0.297		
EF (%)	0.85 (0.51–1.18)	0.009	0.96 (0.65–2.01)	0.237
Hemoglobin (g/dL)	0.89 (0.74–1.43)	0.022	0.88 (0.62–2.41)	0.211
Total cholesterol (mg/dL)	1.09 (0.78–2.67)	0.477		
LDL cholesterol (mg/dL)	1.28 (0.73–2.05)	0.201		
HDL cholesterol (mg/dL)	0.91 (0.69–1.57)	0.286		
Triglycerides (mg/dL)	1.17 (0.82–1.84)	0.366		
ACE/ARB (%)	1.02 (0.45–2.04)	0.527		
Beta blockers (%)	0.97 (0.54–2.36)	0.525		
Statins (%)	0.92 (0.56–2.11)	0.218		
Diuretics (%)	1.03 (0.44–2.11)	0.233		
Albuminuria (%)	1.11 (0.86–2.01)	0.014	1.08 (0.69–3.02)	0.171
Hypotension during PCI (%)	1.21 (1.03–2.13)	0.013	1.18 (0.93–2.57)	0.089
CV > MACD (%)	1.39 (1.05–2.17)	<0.001	1.23 (0.99–2.11)	0.001
Number of diseased vessels (*n*)	1.33 (1.03–2.18)	0.004	1.27 (0.99–2.03)	0.006
eGFR (mL/min/1.73 m^2^)	0.80 (0.37–1.81)	0.189		
HSP27 (µg/L)	0.78 (0.53–1.11)	<0.001	0.81 (0.51–1.37)	0.001

Abbreviations: MI, myocardial infarction; EF, ejection fraction; PCI, percutaneous coronary interventions; CV > MACD, contrast volume > maximum allowed contrast dose; ACE/ARB, angiotensin-converting enzyme inhibitors/angiotensin receptor blockers; HSP27, heat shock protein 27; OR, odds ratio; CI, confidence interval.

## Data Availability

The data used to support the findings of this study are available from the corresponding author upon request.
